# Transketolase Is Identified as a Target of Herbicidal Substance *α*-Terthienyl by Proteomics

**DOI:** 10.3390/toxins10010041

**Published:** 2018-01-12

**Authors:** Bin Zhao, Jingqian Huo, Ning Liu, Jinlin Zhang, Jingao Dong

**Affiliations:** 1 College of plant protection, Agricultural University of Hebei, Baoding 071000, China; bdzhaobin@126.com (B.Z.); huojingqian@163.com (J.H.); 2 College of life science, Agricultural University of Hebei, Baoding 071000, China; lning121@126.com

**Keywords:** *α*-terthienyl, proteomics, herbicidal mechanism, transketolase, The mode of action of plant toxin *α*-terthienyl is identified for the first time, and the target is validated as transketolase by two-dimensional gel electrophoresis. Transketolase is a good candidate target for hercide design.

## Abstract

*α*-terthienyl is a natural phytotoxin isolated originally from *Flaveria bidentis* (L.) Kuntze. The bioassay presented here shows the strong herbicidal activity of *α*-terthienyl on *Digitaria sanguinalis*, *Arabidopsis thaliana* and *Chlamydomonas reinhardtii*. The *α*-terthienyl-induced response of *A. thaliana* at the protein level was analyzed at different times. Changes in the protein expression profiles were analyzed by two-dimensional gel electrophoresis and liquid chromatography tandem mass spectrometry (LC-MS/MS) mass spectrometry. Sixteen protein spots were identified that showed reproducible changes in the expression of at least 2-fold when compared to the control. Among these 16 spots, three were up-regulated and 13 were down-regulated. The decreased expression of several proteins associated with energy production and carbon metabolism suggested that these processes were affected by *α*-terthienyl. To search for the candidate proteins in this screen, *A. thaliana* T-DNA mutants of the candidate proteins were used to test their susceptibility to *α*-terthienyl. Amongst the others, *attkl1*, a mutant of transketolase, exhibited a significantly lower sensitivity to *α*-terthienyl when hit compared with Col-0. Based on the identification of the proteins associated with the response to *α*-terthienyl by proteomics, a candidate target protein transketolase was identified.

## 1. Introduction

Since the initial description of 2,4-dichlorophenoxyacetic acid in 1942, many chemical herbicides have been developed and successfully applied in agriculture. As the mode of action (MoA) for these herbicides has been gradually elucidated, the structure and targets of herbicides have been characterized. Early MoA studies relied on physiological and biochemical technology such as the finding that glyphosate was a potent inhibitor of 5-enolpyruvyl-shikimic acid-3-phosphate synthase [1] or the demonstration that sulfonylurea herbicide was an extremely potent and selective inhibitor of acetolactate synthase [2,3]. The second phase of MoA research began in the 1990s. In this phase, the development of molecular biological techniques allowed the identification of nucleic acid mutations in target-resistant mutants [4] and the production of recombinant proteins for enzyme assays [5,6,7], facilitating MoA identification. In the third phase, ‘omics’ approaches were used to determine the mode of action of phytotoxins with previously unknown modes of action [8]. Including inquiries to the most fundamental MoAs to phenotypic responses, ‘omics’ approaches include transcriptomics, proteomics, metabolomics, physionomics, and phenomics. These strategies can be used to generate an abundance of data for compounds with known modes of action. Then, the response profile to a compound with an unknown mode of action is produced and compared with those for a known mode of action. As more plants have been sequenced, these types of ‘omics’ approaches have been used widely. 

Proteomics is the large-scale study of proteins, particularly function and structure. Responses to external stimuli can be reflected at the protein level and analyzed by two-dimensional gel electrophoresis coupled with mass spectrometry. Currently, this approach is increasingly applied in biological science. Herbicides such as natural phytotoxins are expected to cause variations in protein expression level. 

**Figure 1:**
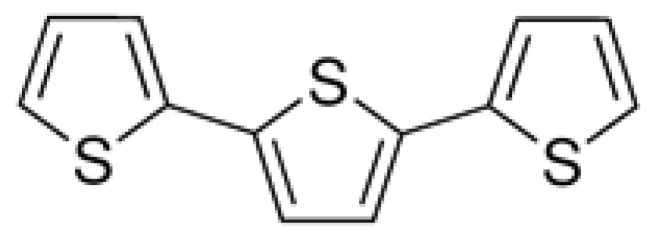
Structural formula of *α*-terthienyl.

Our previous studies have showed that 9.25 mg *α*-terthienyl could be extracted from 100 g root of *Flaveria bidentis* (L.) Kuntze [9]. The α-terthienyl structural formula is shown in Figure 1 [10]. Some studies have demonstrated that *α*-terthienyl has significant photoactivatable insecticidal activity in daylight or 300–400 nm UV light conditions [11,12,13]. *α*-terthienyl is a type-II photosensitizer that is toxic toward a variety of insects, nematodes, micro-organisms, human blood cells and plants [14,15]. For example, low concentrations of *α*-terthienyl in combination with UVA radiation can damage chloroplast membranes, interfere with enzymes in the Calvin Cycle, and inhibit photosynthetic CO_2_ fixation with light activation [16]. However, as an herbicidal activity component, *α*-terthienyl has an unknown mode of action, identification the new target gene/protein will benefit pesticide creation. In this study, using a proteomic approach, we identified differentially expressed proteins (DEPs) that suggests the MoA of *α*-terthienyl and found new herbicide targets as well.

## 2. Results

### 2.1. Herbicidal Activity of α-Terthienyl on Plants

In this study, the median inhibitory concentration (IC_50_) was determined for *D. sanguinalis*, *A. thaliana* and *C. reinhardtii* as shown in Table 1. *C. reinhardtii* is a unicellular green alga, which has served as a model in molecular biology and photosynthesis research [17] including studies for various herbicides [18,19]. *α*-terthienyl has a lower IC_50_ relative to atrazine for all plants tested. The IC_50_ of *α*-terthienyl and atrazine in *C. reinhardtii* is 1.10 and 2.26 (mg/L), respectively. *α*-terthienyl has a higher herbicidal activity than atrazine. The activity of *α*-terthienyl was also tested in dark and light for *C. reinhardti*, as shown in the Figure S1, where the result shows that *α*-terthienyl still retained activity in the darkness. Furthermore, Campbell [16] and Brennan [20] also reported the activity of *α*-terthienyl on plants in dark, and Gommers [21] reported that it had a high activity on nematodes. It does seem that *α*-terthienyl is capable of exerting toxic effects in the absence of light.

### 2.2. Sample Extraction and Two-Dimensional Gel Electrophoresis

**Figure 2:**
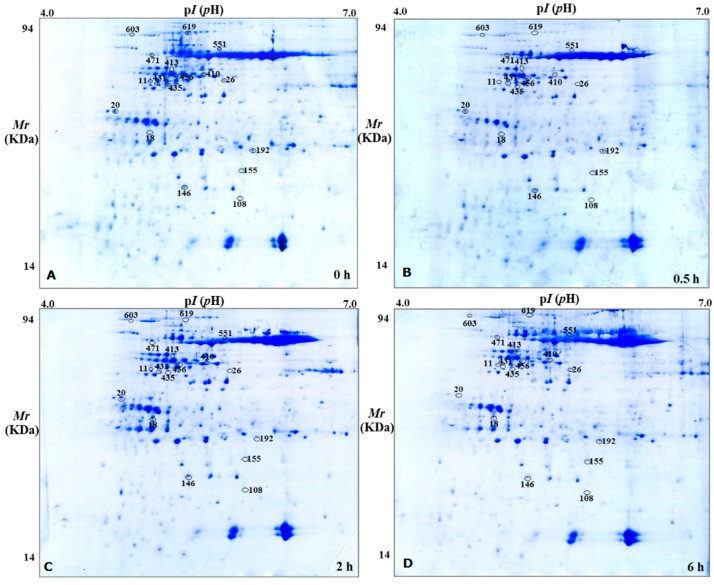
Distribution of expressed proteins (DEPs) in PAGE gel. (**A**) Treated by *α*-terthienyl for 0 h; (**B**) treated by *α*-terthienyl for 0.5 h; (**C**) treated by *α*-terthienyl for 2 h; (**D**) treated by *α*-terthienyl for 6 h.

Treatment time with *α*-terthienyl is closely related with its effect as measured by two-dimensional gel electrophoresis. *A. thaliana* was examined after treatment with *α*-terthienyl at 0.5 h, 2 h, and 6 h by 2-DE. The high-resolution 2-DE gel pattern with a pI ranging from 4–7 was detected by coomassie brilliant blue (CBB) staining (Figure 2). More than 700 protein spots were detected in each CBB-stained gel by ImageMaster software. There were 848, 781, 708 and 768 protein spots detected after *α*-terthienyl treatment at 0 h, 0.5 h, 2 h, and 6 h respectively (gels shown in Figure 2A–D, respectively). Normalized protein spot volumes were determined for each 2-DE gel, and the number of proteins was inferred using a calibration curve based on different quantities of protein standards [22]. 

### 2.3. Mass Spectrometry and Analysis of DEPs

**Figure 3:**
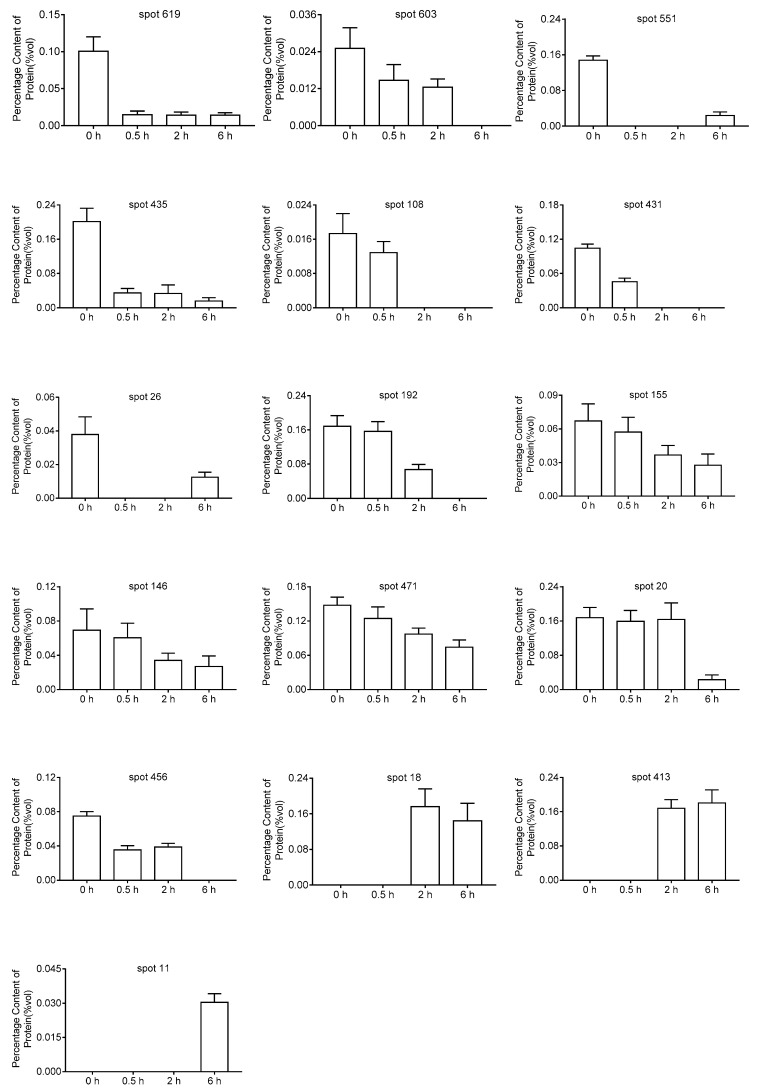
DEPs regulated by *α*-terthienyl.

In this study, protein spots with at least 2-fold changes had excised from 2-DE and identified by Mass Spectrometry. Q Exactive, with high resolution, good stability, high sensitivity and rapid analysis was used to identify the protein. Through LC-MS/MS, 13 down-regulated proteins and three up-regulated proteins were identified in this study, which changed steadily after the treatment with *α*-terthienyl. These proteins were involved in metabolism, transcription, translation, stress, and other processes. The expression of many kinds of proteins was inhibited after the treatment of *α*-terthienyl such as spots 619, 603, 551, 435, 108, 431, 26, 192, 155, 471, 456, and 20, while the expression of some proteins like spots 413, 18, and 11 were increased. From these 16 spots, two of them were identified the same one: spot 435 and 456. The protein expression level and description of DEPs are shown in Figure 3 and Table 2, respectively.

### 2.4. The Gene Ontology and Protein-Protein Interaction Networks Analysis of DEPs

**Figure 4:**
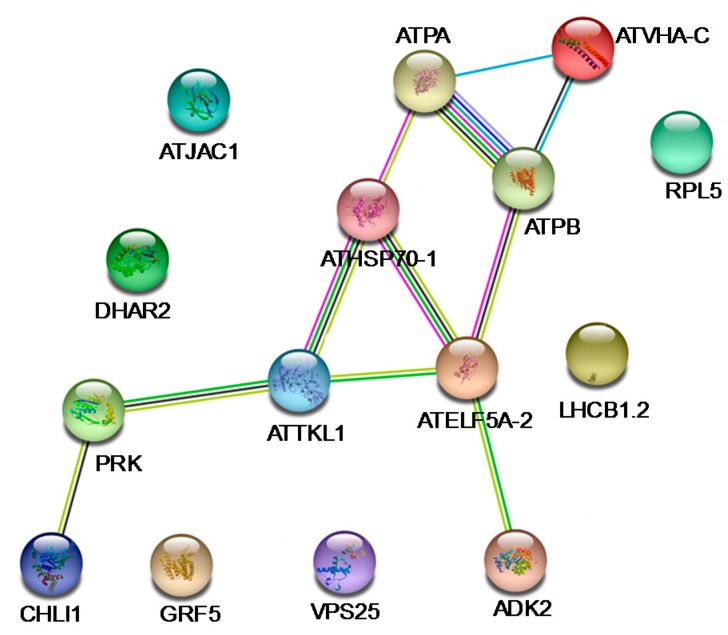
Protein-protein interaction regulatory network of DEPs. The regulatory network of DEPs was done using String software. Differentially regulatory network was represented by node. Different colors of lines represent different evidences for the predicted functional relationship between proteins; red line, gene fusions; dark blue line, gene co-occurrence; black line, co-expression; yellow line, text mining; green line, gene neighborhood; light blue line, database; and pink line, experimentally determined.

The DEPs in Gene Ontology (GO) were mainly involved in the metabolic process, cellular components and catalytic activity in molecular functions (Figure S2). Therefore, we deem that *α*-terthienyl could affect the expression of related enzymes in the metabolic process resulting in plant death. All those DEPs identified in the current study were submitted to STRING to assess the protein-protein interaction (PPI) networks (Figure 4). The network contained 15 nodes and 11 DEPs with an average node degree of 1.47 and a clustering coefficient of 0.3. The PPI enrichment p-value was equal to 0.00322 and 0.000388. The results showed that the ATTKL1 had a complex regulatory relationship with ATPB, PRK, ATELF5A-2 and ATHSP70-1, which participated in translation, energy metabolism, and defense response. Taken together, these results provide further evidence to support the mode of action for *α*-terthienyl.

### 2.5. Quantitative Real-Time PCR Analysis for DEPs

**Figure 5:**
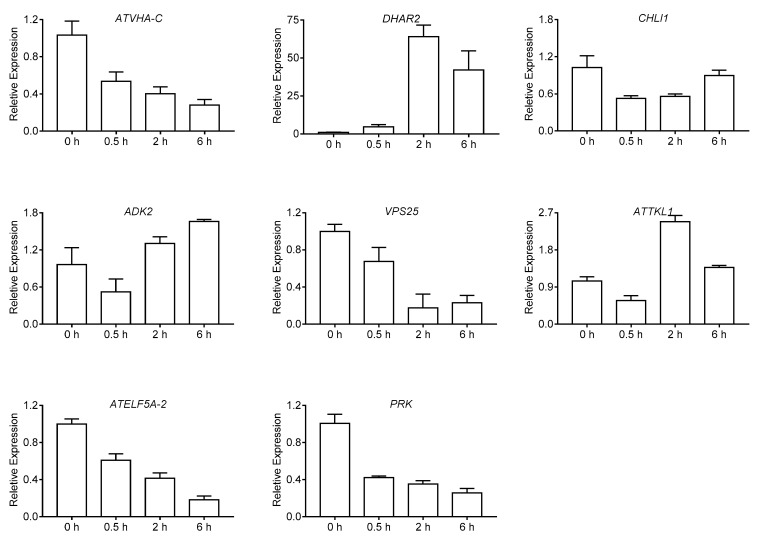
The identification of different expression proteins by Quantitative Real-Time PCR (Q-PCR).

After *α*-terthienyl treatment, the RNA and protein expression of *ATELF5A-2*, *PRK*, *VPS25* and *ATVHA-C* were decreased, the RNA and protein expression of *ADK2* and *DHAR2* were increased as shown in Figure 5. However, the measurements of RNA and protein expression of the *ATTKL1* gene were not in accordance with each other. The ATTKL1 protein decreased, but the Quantitative Real-Time PCR (Q-PCR) analysis indicated a higher gene expression at the transcriptional level. This result may be explained by inhibition of protein activity caused by the binding of *α*-terthienyl to the ATTKL1 protein, followed by an increase in *ATTKL1* gene expression to compensate for the loss of protein activity (Figure 5).

### 2.6. Transketolase Is One of Target Proteins Which Respond to α-Terthienyl

**Figure 6:**
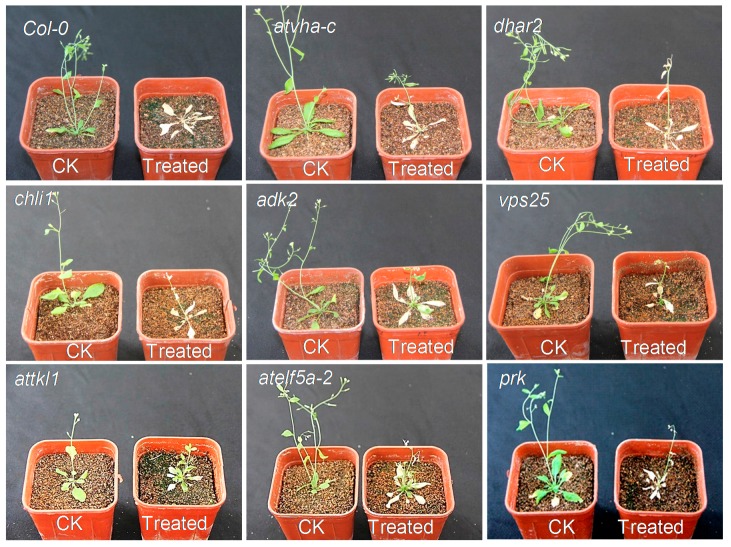
The sensitivity of *Arabidopsis* mutant to *α*-terthienyl.

**Figure 7:**
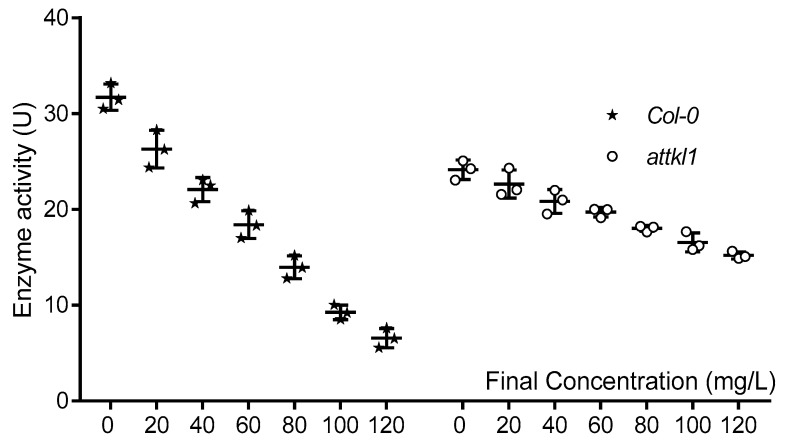
The enzyme activity of *Col-0* and *attkl1* which was treated by *α*-terthienyl.

The homozygous of mutants *prk*, *attkl1, chli1* and at*vha-c* were indentified by Three Primers-PCR (TP-PCR, Figure S3). Those mutants were tested by drug sensitivity. The results showed that the *Col-0* and the mutants exhibited wilting death except *attkl1* (SAIL_58D02) (Figure 6). The *attkl1* had short stems and leaves when compared with the wild type, but after the treatments, it did not exhibit wilting death phenomenon, which presumably was due to the insertion of T-DNA. The T-DNA was inserted in the first exon of *attkl1*, the gene structure of which is shown in Figure S4. The primary structure of ATTKL1 protein will be frameshift or insertion mutated due to the insertion of T-DNA, which can decrease the affinity between the small molecules compounds and the protein. Hence, we inferred that *α*-terthienyl could interact with ATTKL1, when the gene was mutated, following with the change of encoded protein leading to a failure combination of *α*-terthienyl with the protein ATTKL1. In summary, the ATTKL1 protein was considered as a candidate protein, and was analyzed and verified in the next step. It plays a central role in the Calvin cycle of plant photosynthesis, its activity is a limiting factor of the photosynthetic rate. A slight decline of transketolase activity can reduce the plant growth rate and suppress the metabolites of aromatic amino acids and phenylalanine. Indeed, studies of transketolase mutant in tobacco have shown that small decreases in TKL can cause erythrose 4-phosphate levels to reduce, leading to photosynthesis inhibition and a significant decrease in soluble phenylpropanoids and aromatic amino acids [23]. Transketolase activity was tested with *α*-terthienyl of different concentrations, and transketolase activity decreased gradually with the concentration increasing of *α*-terthienyl (Figure 7). Results showed that transketolase activity in the wild type strain was more sensitive than that of the mutant. Indeed, there were two highly conserved paralogues of TKL, ATTKL1 and ATTKL2 in *A. thaliana*, which were predicted to reside in the chloroplast [24]. The homology of ATTKL1 and ATTKL2 were up to 88.26% (data not shown), both contained 740 amino acids. Nevertheless, only ATTKL1 was expressed in the highest levels in photosynthetic tissue ubiquitously. Unlike ATTKL1, ATTKL2 was expressed in development playing an important role in carbon allocation [24]. This caused the binding of *α*-terthienyl to diminish while some enzymatic activity retained in *attkl1*.

## 3. Discussion

### 3.1. Proteomic Approach is a Powerful Tool for Identification the Mode of Action of Herbicide

There are many reports of the identification of DEGs with Proteomic approach. Nestler et al. [25] tested the proteome of the green alga *C. reinhardtii* after treatment with low and high doses of norflurazon and diuron. Approximately 149–254 significantly altered proteins were found, and some were associated with specific modes of action. For example, the 1-deoxy-d-xylulose 5-phosphate synthase of the plastidic isoprenoid pathway were upregulated when *C. reinhardtii* was treated with norflurazon. For diuron, the target PS II-D1 protein was changed. Holmes et al. [26] used two dimensional gels to analyze the roots and meristem tissues of *Medicago truncatula* treated with acetolactate synthase (ALS) inhibiting herbicides. Eighty-one protein spots and 51 protein spots were changed in meristematic and nonmeristematic tissues, respectively.

In this study, two-dimensional gels were used to identify DEPs with different treatment times of *α*-terthienyl on *A. thaliana*. Some proteins which play important role in plants developments and growth were identified. Such as Spot ID 619 was identified as transketolase (ATTKL1), and was down-regulated more than 3-fold in all treatments. The amount of protein was rapidly reduced when *A. thaliana* was treated with *α*-terthienyl. Transketolase, presented in all organisms, is a key enzyme in the pentose phosphate pathway and the Calvin cycle of photosynthesis [27,28]. Thus, the transketolase plays an important role in plant defense and growth, making it a good herbicide target. At the 0.5 h post treatment with *α*-terthienyl, the transketolase content rapidly decreased by approximately 90%. The low level of transketolase should limit the efficiency of pentose phosphate pathway and calvin cycle, hindering the photosynthesis rate and resulting in plant death; Another is Spot 431, which was identified as magnesium-chelatase subunit ChlI-1, an enzyme involved in chlorophyll biosynthesis [29,30], catalyzing the insertion of magnesium ion into protoporphyrin IX to yield Mg-protoporphyrin IX. The reaction takes place in two steps with an ATP-dependent activation followed by an ATP-dependent chelation step. ChlI-1 possesses high affinity for ATP and may play a major role in chlorophyll biosynthesis. The plant could be etiolated when ChlI-1 levels were decreased or eliminated [31]; the other is Phosphoribulokinase (Spot 435) which catalyzes the ATP-dependent phosphorylation of ribulose-5-phosphate to ribulose-1,5-phosphate, a carbonate receptor of light synthesis and a key step in the pentose phosphate pathway, where carbon dioxide is assimilated by autotrophic organisms [32]. The phosphoribulokinase was down-regulated after *α*-terthienyl treatment. Down-regulation of phosphoribulokinase can reduce the production of ribulose-1,5-phosphate, thus affecting photosynthesis and resulting in plant death. 

Although we identified many proteins strongly affected by *α*-terthienyl though 2-DE, proteomic analysis was limited if the transcript information of gene (or protein) was not available in Genebank to identify the sequence of peptides. Another drawback is that proteomic approaches are limited to soluble proteins [33]. Recently, gel-free proteomic methods have been developed out isobaric tags for relative and absolute quantitation (iTRAG), and other protein tagging methods integrated with LC-MS/MS are likely to be powerful tools to overcome the limits of 2-D gel-based proteomics [34].

### 3.2. α-Terthienyl Can Inhibit Photosynthesis though Transketolase

It is generally acknowledged that, *α*-terthienyl has been developed as an effective photoactivated insecticide against pests, which can act on the cuticle enzymes, the midgut membranes, the neuromuscular sheath, and so on [35]. When the ovarian cells were exposed to *α*-terthienyl, it changed the generation of oxidative stress, caused nonselective DNA damage which could increase apoptosis, G1 arrest and decrease cell population in S phase [36]. Another well-known example of toxicity in *α*-terthienyl dependent on light is the killing of endoparasitic plant nematodes in the roots of Asteraceae [21]. Cilento proposed that the excited-state species generated as products of certain enzymatic reactions were capable of transferring excitation energy to other molecules such as chlorophyll and xanthene dyes. This indicates that it has a photochemistry mode and a photobiology mode in mode of action [37,38,39,40]. In addition, *α*-terthienyl can exert toxic effects when in the absence of light, and it has some activity on plants and nematodes [41]. Furthermore, Brennan reported that *α*-terthienyl could interfere the enzymes in Calvin cycle and inhibit photosynthetic CO_2_ fixation [16]. 

In this study, T-DNA mutants of *A. thaliana* were used for screening sensitive strains. Since the gene coding region is inserted by a long T-DNA fragment, it will inevitably lead to a big change in protein structure, which to a great extent may affect the binding of small molecules and proteins. *Attkl1*, a mutant of transketolase, was identified in this way. In our previous study, the molecular docking of ATTKL1 and *α*-terthienyl was performed by computer simulation, where the results confirmed that *α*-terthienyl was perfectly combined with ATTKL1. Furthermore, the fluorescence quenching spectroscopy also verified their combination [42]. We identified the key amino acids involved in the interaction between transketolase and *α*-terthienyl. According to our previous study and the UniProt Archive database, the amino acid binding sites for ATTKL1 were His^143^, Gly^234^, Asn^263^, Arg^434^, Ser^461^, Gln^488^, Phe^515^, His^539^, Asp^547^ and Arg^598^ (Figure S5). The catalytic amino acids were His^103^ and His^340^. Our previous study showed that ATTKL1 can bind with *α*-terthienyl using binding sites His^143^, Gly^234^, and the catalytic sites His^103^ and His^340^. According to the literature and our research results, we hypothesized that when the plants come into contact with *α*-terthienyl, which can bind to the transketolase in the plant; it inhibited the active center of transketolase, rendering it unable to transfer Ketol to phosphate aldose, resulting in blocking of photosynthesis up to plant death.

All these results indicated that *α*-terthienyl may firstly affect the activity of transketolase. Effects induced by *α*-terthienyl will be studied in future studies, which will provide the foundation for characterizing the herbicidal mechanism and mode of action of *α*-terthienyl. Additionally, this will provide a reference for discoveries of target sites of new pesticides. 

## 4. Conclusions

This study analyzed the DEPs of *A. thaliana* after exposure to *α*-terthienyl at different times, using two-dimensional gel electrophoresis. *α*-terthienyl is a herbicidal active substance isolated from the *Flaveria bidentis* (L.) Kuntze. The results showed that 16 DEPs were identified. Among these proteins, 3 were up-regulated and 13 were down-regulated. Many of them were involved in photosynthesis respiration, energy synthesis, and metabolites synthesis. The accuracy of the protein data was verified by real-time quantitative PCR. The candidate protein ATTKL1 was found through the susceptibility testing of *α*-terthienyl in *A. thaliana* T-DNA mutants. Based on the differential proteomic analyses and the relevant previously released data, we propose that the candidate protein transketolase could interact with *α*-terthienyl. The results are of great theoretical significance for the creation of new pesticides.

## 5. Materials and Methods

### 5.1. The Herbicidal Bioassay of α-Terthienyl

The effect of *α*-terthienyl on *D*. *sanguinalis*, *A. thaliana* and *C. reinhardtii* was determined with the foliar treatment [43]. 

### 5.2. Plant Material and Treatments

*A. thaliana* Columbia (Col-0) was kindly provided by Dr. Xia of the Hong Kong Baptist University. Two-week-old *A. thaliana* plants were treated with *α*-terthienyl at different times (0 h, 0.5 h, 2 h, and 6 h); the *A. thaliana* treated at 0 h was used as a control, the aerial part samples were collected and kept at −80 °C and used for 2-DE. 

### 5.3. 2-DE and Data Analysis

*A. thaliana* proteins were extracted by trichloroacetic acid acetone (TCA-acetone) precipitation. The aerial parts of *A. thaliana* were triturated in liquid nitrogen with 10% polyvinylpolypyrrolidone (PVPP). The homogenates were lysed with precooled acetone (with 10% TCA) in a centrifuge tube at −20 °C. The samples were centrifuged at 6000 g for 10 min at 4 °C. Next, the pelleted material after centrifugation was rinsed twice with acetone and then sonicated for 5 min in 1 mM Phenylmethanesulfonyl fluoride (PMSF), 2 mM ethylenediaminetetraacetic acid (EDTA), and 10 mM Dithiothreitol (DTT). The protein fractions were then obtained by centrifugation at 15,000 *g* for 20 min. The protein concentration was determined using the RC-DC Protein Assay kit (Bio-Rad, Hercules, CA, USA) with BSA as the standard.

Twenty-four cm Immobiline DryStrip Gels (pH 4–7, 130 × 3 × 0.5 mm; Bio-Red) were used for isoelectric focusing with 700 μg of total proteins (mixed in 2% CHAPS, 18 mM DTT, 0.8% IPG buffer with 350 µL rehydration buffer). Rehydration and focusing were performed in an Ettan IPGphor II (GE Healthcare, LON, UK) at room temperature according to the following program: rehydration 8 h; 50 V for 8 h; 500 V for 1 h; 1000 V for 1 h; 8000 V for 2 h; and 8000 V in 75,000 Vh. The immobilized pH gradient (IPG) strip was removed to a hydration disc after isoelectric focusing (IEF), and equilibrium and SDS-PAGE were performed according to the modified method of Giavalisco and Kamo [44]. The two-dimensional electrophoresis gels were stained with Coomassie Brilliant Blue as described in the Neuhoff method [45]. Each sample was analyzed with data from at least three experimental replicates. 

### 5.4. Image Acquisition and Data Analysis

2-DE gels were stained by Coomassie Brilliant Blue (CBB) to image and analyze the data. Digitalized images of CBB-stained 2-DE gels were made using a high-resolution scanner (UMAX Powerlook 2100XL-USB, UMAX, Houston, TX, USA). The protein spots with increased or decreased intensity were detected and quantified with the ImageMaster software (ImageMaster 2D platinum 5.0; GE Healthcare, LON, UK) based on their relative volume. The amount of the protein spot was expressed as the volume of the spot, which was defined as the sum of the intensities of all the pixels of the spot. To compensate for subtle differences in sample loading, gel staining, and destaining, the volume of each spot was normalized as a percentage of the total volume of all of the spots present in the gel. The relative molecular mass (*M*r) and isoelectric point (p*I*) of each protein were determined using 2-D SDS-PAGE standards after detection and matched by automated and manual editing. The DEPs which have at least 2-fold change were excised from the gels. Three replicates from three independent biological extracts were used for analysis. 

### 5.5. Proteins Identified by Mass Spectrometry

Gel spots from 2-DE were carefully excised, successively destained, and dehydrated with destaining solution (50% methyl cyanides and 25 mM ammonium bicarbonate). The proteins were incubated with 10 mM DTT in 25 mM ammonium bicarbonate at 56 °C for 1 h and alkylated with 55 mM iodoacetamide in 25 mM ammonium bicarbonate at room temperature in dark for 45 min, followed by the removal of surplus iodoacetamide. The gels were then washed twice in 25 mM ammonium bicarbonate for 10 min. Finally, the gel pieces were thoroughly washed and the methyl cyanides were removed in a Speedvac. The proteins were digested in modified trypsin solution (67 μg/mL trypsin in 25 mM ammonium bicarbonate) though incubation overnight at 37 °C. The gel pieces were extracted with 1 mL destaining solution for 30 min and dehydrated with 1 mL methyl cyanides. For LC-MS-MS, freeze-dried digestive products were dissolved in 0.1% formic acid. Digested peptides were separated on octadecylsilyl reverse-phase capillary columns (5 μm, 150 Å, Venusil × BPC; Agela Technologies, Tianjin, China) using the Ultimate 3000 LC system (Dionex, Sunnyvale, CA, USA). Flow was maintained at 0.4 μL/min. Solvents A and B were 99.9/0.1 water/formic acid and 99.9/0.1 acetonitrile/formic acid, respectively, and were used for peptide elution. All digested peptides were analyzed by Q Exactive (Thermo Scientific, Waltham, MA, USA). Detailed instrument parameters were as follows, positive ion for ion mode, 70 K resolution for the full MS scans, 17.5 K resolution for the high energy collisional dissociation (HCD) MS/MS scans, and the mass spectrometer was operated under 1.8 kV accelerating voltage in the reflectron mode with a *m*/*z* range 600–4000. All MS and MS/MS spectra were obtained in profile mode. Raw LC-MS-MS data files were processed into peak lists in MGF format using Thermo Scientific Proteome Discoverer software version 1.3 (Thermo Scientific, Waltham, MA, USA). The results of the MGF files were searched using MASCOT (Matrix Science, Columbia, SC, USA) against the *A. thaliana* database (541,954 sequences; 192,668,437 residues). These sequences of the DEPs were matched to cellular components, molecular functions, and biological processes as classified in the GO database.

### 5.6. RNA Extraction and Q-PCR Analysis

The RNA of *A. thaliana* which has same treatment as protein extraction was isolated by using E.Z.N.A.^®^ Plant RNA Kit (Omega, Irving, TX, USA), and mRNA was reversely transcribed into cDNA. Q-PCR was performed as the conventional method. The primers used for Q-PCR are shown in Table S1. 

### 5.7. The Sensitivity Test of α-Terthienyl in T-DNA Mutants

The T-DNA mutants associated with the DEPs were obtained from the *A. thaliana* Information Resource (TAIR) center. Those homozygous mutants were identified by TP-PCR [46] (the primer sequences are presented in Table S1) and cultured in vitro. The homozygous segregants mutants *prk*, *attkl1*, *chli1* and at*vha-c* were identified by TP-PCR. However, *vps25*, *adk2*, *atelf5a-2* and *dhar2* were not indentified as they were belonged to homozygous lines. After four weeks, the *A. thaliana* seedlings were treated with 100 mg/L *α*-thiophene or methanol to confirm the effects of drug treatment.

### 5.8. Enzyme Activity Assay of Transketolase

Plant tissue was ground in liquid nitrogen, then mixed with reaction liquid of 50 mmol/L Tris-HCl solution (pH 7.8), 0.2 mmol/L nicotinamide adenine dinucleotide, 1 mmol/L thiamine pyrophosphate, 2 mmol/L Ribose 5-phosphate, 10 mmol/L MgCl_2_, and 2 mmol/L xylulose-5-phosphate. The enzymatic reaction was initiated by addition of 9 U of triosephosphate isomerase and 3 U of alpha glycerol dehydrogenase and after two minutes, the absorbance was measured at 340 nm. The amount of enzyme required for the conversion of 1 μmol NADH in 1 min at 30 °C was defined as a dynamic unit (U).

## Figures and Tables

**Table 1:** The Herbicidal activity of *α*-terthienyl on plants.

Species/Treatment	Median Inhibitory Concentration (mg/L)	Toxicity Regression Equations	*R*^2^
*D*. *sanguinalis/α*-terthienyl	22.03	*y* = 4.30 + 0.51*x*	0.9209
*D*. *sanguinalis/*atrazine	32.95	*y* = −3.09 + 2.02*x*	0.9162
*A. thaliana/α*-terthienyl	29.64	*y* = 4.30 + 0.49*x*	0.9199
*A. thaliana/*atrazine	44.57	*y* = 4.01 + 0.63*x*	0.9559
*C. reinhardti/α*-terthienyl	1.10	*y* = 4.97 + 0.65*x*	0.9597
*C. reinhardti/*atrazine	2.26	*y* = 4.78 + 0.63*x*	0.9778

**Table 2:** DEPs of *α*-terthienyl-induced in *A. thaliana* identified by LC-MS/MS.

Spot ID	Biological Process	Protein Name	Gene No.	Gene Name	Coverage (%)	p*I*/*M*r Experimental	p*I*/*M*r Theoretical	Different Expression Protein Detected by 2-DE			
								0 h	0.5 h	2 h	6 h
619	Carbohydrate metabolism	Transketolase-1	AT3G60750	ATTKL1	74	5.42/81	5.94/80	〇	↓	↓	↓
603	defense response	heat shock cognate protein 70-1	AT5G02500	ATHSP70-1	71	4.95/77	5.03/72	〇	↓	↓	↓
551	Energy metabolism	ATP synthase subunit beta	ATCG00480	ATPB	88	5.64/60	5.38/54	〇	↓	↓	↓
435	reductive pentose-phosphate cycle	Phosphoribulokinase	AT1G32060	PRK	71	5.21/41	5.71/44	〇	↓	↓	↓
108	Nucleotide and amino acid translation	Eukaryotic translation initiation factor 5A-2	AT1G26630	ATELF5A-2	72	5.83/18	5.55/17	〇	≡	↓	↓
431	Photosynthesis	Magnesium-chelatase subunit ChlI-1	AT4G18480	CHLI1	66	5.21/46.5	6.08/46.5	〇	↓	↓	↓
26	Energy metabolism	V-type proton ATPase subunit C	AT1G12840	ATVHA-C	81	5.68/41	5.4/42	〇	≡	↓	↓
192	Response to Stress	Glutathione S-transferase DHAR2	AT1G75270	DHAR2	81	5.97/25	5.79/24	〇	≡	↓	↓
155	translation	60S ribosomal protein L5	ATMG00210	RPL5	32	5.90/22	5.6/21	〇	≡	↓	↓
146	Protein transport	Vacuolar protein sorting-associated protein 25	AT4G19003	VPS25	26	5.30/20	5.48/20	〇	≡	↓	↓
471	Energy metabolism	ATP synthase subunit alpha	ATCG00120	ATPA	76	5.20/60	5.19/55	〇	≡	↓	↓
20	response to cadmium ion	14-3-3-like protein GF14 upsilon	AT5G16050	GRF5	78	4.72/32	4.73/30	〇	≡	↓	↓
456	reductive pentose-phosphate cycle	Phosphoribulokinase	AT1G32060	PRK	62	5.34/44	5.71/44	〇	≡	≡	↓
18	Carbohydrate metabolism	Chlorophyll a-b binding protein 2	AT1G29910	LHCB1.2	44	5.02/29	5.29/28	〇	≡	≡	↑
413	Response to Stress	Myrosinase-binding protein	AT3G16470	ATJAC1	69	5.26/48	5.12/48	〇	≡	↑	↑
11	AMP salvage	Adenosine kinase 2	AT5G03300	ADK2	68	5.01/43	5.14/38	〇	≡	≡	↑

## Supplementary Materials

The following are available online at www.mdpi.com/2072-6651/10/1/41/s1, Figure S1: The bioactivity of *α*-terthienyl in light and darkness, Figure S2: The GO analysis of Differential expression proteins regulated by *α*-terthienyl, Figure S3: Homozygous mutant identification, Figure S4: The gene structure of SAIL_58_D02, Figure S5: The analysis of key amino acid sites, Table S1: The primers for identification.
