# Supplementary material for: Transketolase Is Identified as a Target of Herbicidal Substance α-Terthienyl by Proteomics
Source: Toxins (Basel). 2018 Jan 12;10(1):41. doi: 10.3390/toxins10010041 (PMC5793128; doi:10.3390/toxins10010041)
Supplement: Supplementary file 1 [file toxins-10-00041-s001.pdf]

Supplementary Materials: Transketolase is Identified as a Target of Herbicidal Substance α-Terthienyl by Proteomics

Bin Zhao, Jingqian Huo, Ning Liu, Jinlin Zhang and Jingao Dong

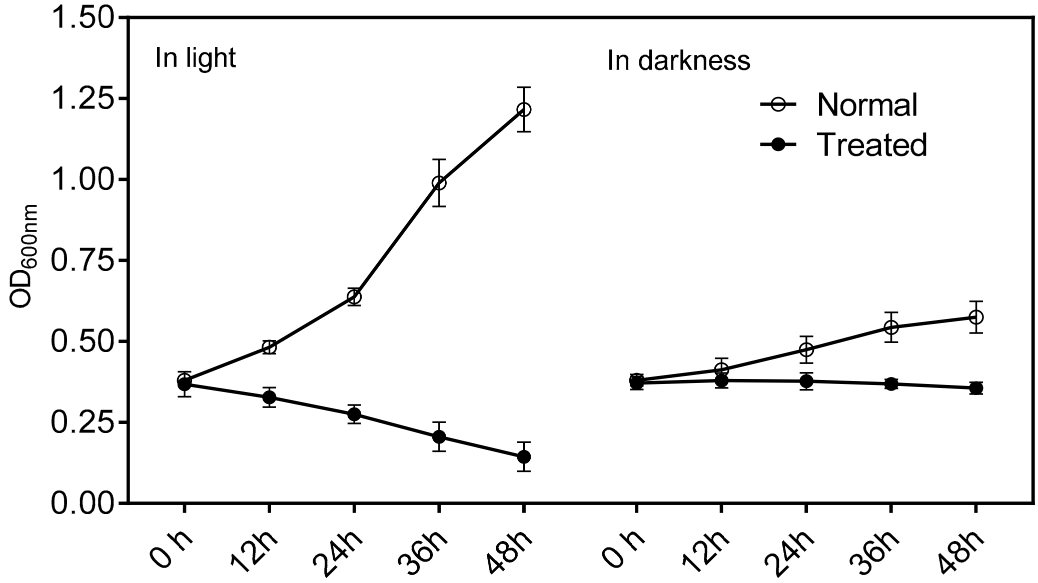

Figure S1. The bioactivity of α-terthienyl in light and darkness.

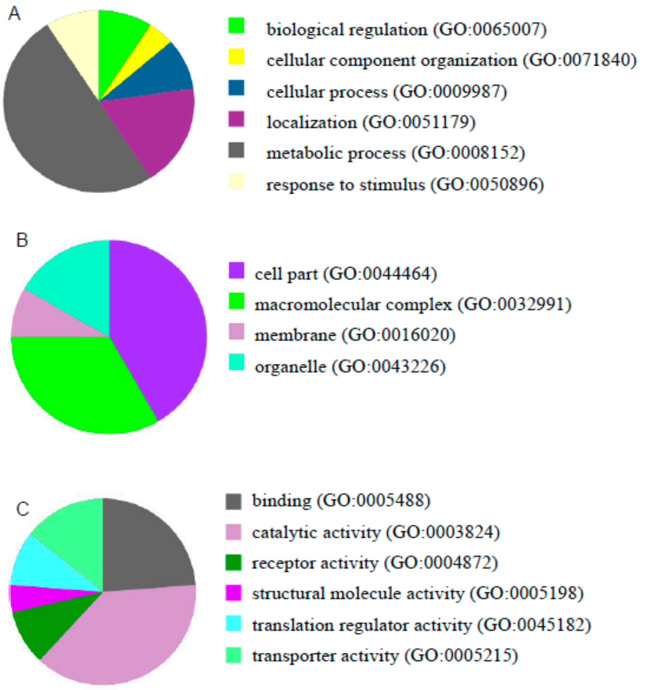

Figure S2. The GO analysis of Differential expression proteins regulated by α-terthienyl. (**A**) Biological processes; (**B**) cellular components; (**C**) molecular functions.

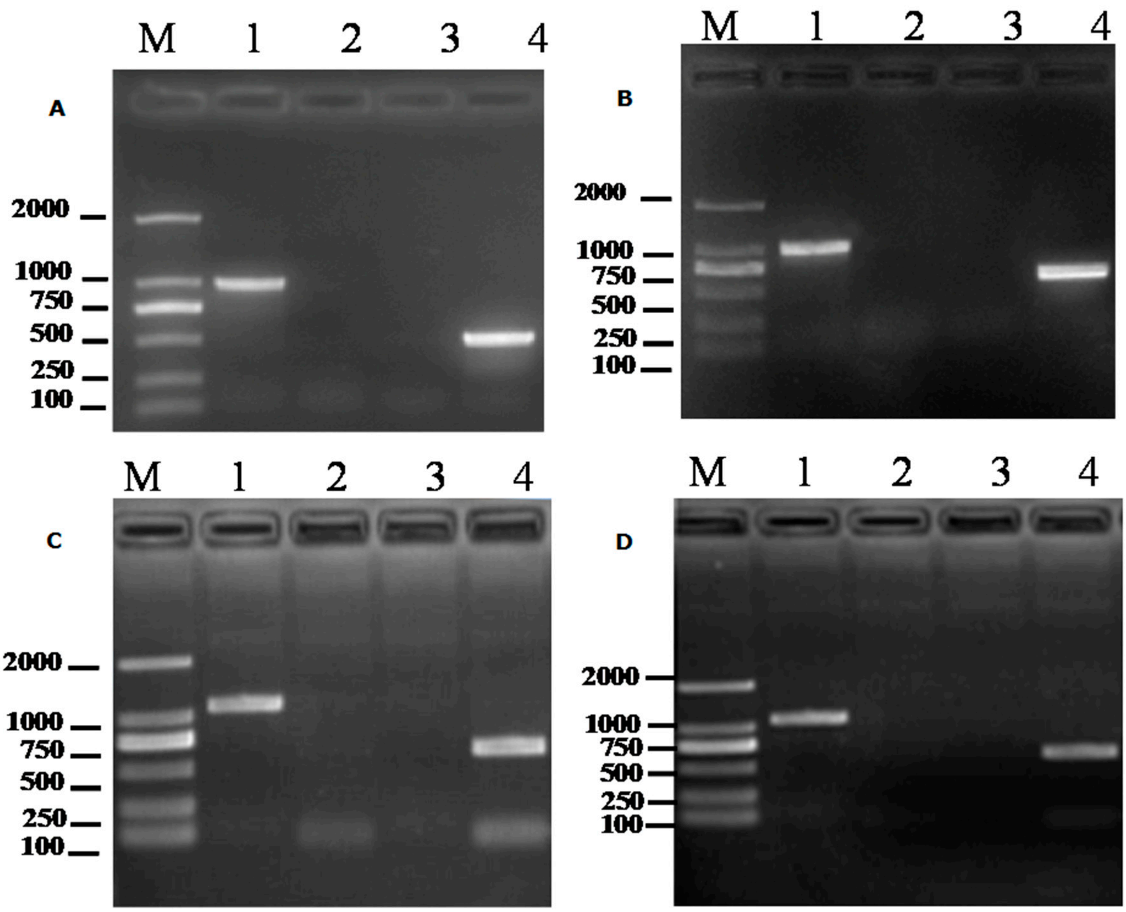

Figure S3. Homozygous mutant identification. The homozygous mutant was amplified by using primers LP and RP, wild type strains can be amplified by the corresponding bands, but not amplified in the mutant; gene amplified by using the primers of LP or RP and LBb1 primers on T-DNA, mutant strains can be amplified by the corresponding bands, but not in the wild type was obtained. *Chli1* homozygous mutant (amplified band size of 996 BP, LP and RP primers BP and RP primers amplified band size was about 500 BP, **A**), *atvha-c* homozygous mutant (amplified band size is 1071 BP, LP and RP primers BP and RP primers amplified bands of size 700 BP, **B**), *attkl1* homozygous mutant, (the size of amplified bands was 996 BP, LP and RP primers BP and RP primers amplified band size was about 500 BP, **C**), *prk* homozygous mutant (amplified band size is 1048 BP, LP and RP primers BP and RP primers amplified band size is about 700 BP, **D**). 1. Col-0, LP+RP; 2. Col-0, BP+RP/LP; 3. homozygote, LP+RP; 4. homozygote, BP+RP/LP；M，DL2000.

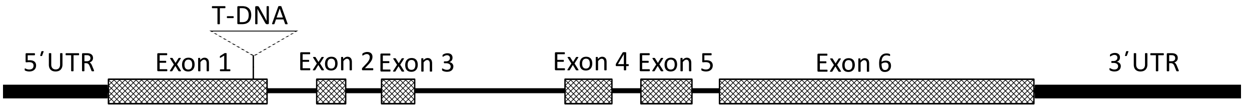

Figure S4. The gene structure of SAIL_58_D02.

```
        10         20         30         40         50         60
MASTSSLALS QALLARAISH HGSDQRGSLP AFSGLKSTGS RASASSRRRI AQSMTKNRSL
        70         80         90        100        110        120
RPLVRAAAVE TVEPTTDSSI VDKSVNSIRF LAIDAVEKAK SGHPGLPMGC APMAHILYDE
                                              *
                                              #
       130        140        150        160        170        180
VMRYNPKNPY WFNRDRFVLS AGHGCMLLYA LLHLAGYDSV QEEDLKQFRQ WGSKTPGHPE
                        *
       190        200        210        220        230        240
NFETPGIEVT TGPLGQGIAN AVGLALAEKH LAARFNKPDA EVVDHYTYAI LGDGCQMEGI
                                                         +*
       250        260        270        280        290        300
SNEACSLAGH WGLGKLIAFY DDNHISIDGD TEIAFTENVD QRFEALGWHV IWVKNGNTGY
                        *
                        + +
       310        320        330        340        350        360
DEIRAAIKEA KTVTDKPTLI KVTTTIGYGS PNKANSYSVH GAALGEKEVE ATRNNLGWPY
                                          *
                                          #
       370        380        390        400        410        420
EPFQVPDDVK SHWSRHTPEG ATLESDWSAK FAAYEKKYPE EASELKSIIT GELPAGWEKA

       430        440        450        460        470        480
LPTYTPESPG DATRNLSQQC LNALAKVVPG FLGGSADLAS SNMTLLKAFG DFQKATPEER
              *                             *
       490        500        510        520        530        540
NLRFGVREHG MGAICNGIAL HSPGLIPYCA TFFVFTDYMR GAMRISALSE AGVIYVMTHD
       *                             *                         *
       550        560        570        580        590        600
SIGLGEDGPT HQPIEHIASF RAMPNTLMFR PADGNETAGA YKIAVTKRKT PSILALSRQK
      *                                                       *
       610        620        630        640        650        660
LPHLPGTSIE GVEKGGYTIS DDSSGNKPDV ILIGTGSELE IAAQAAEVLR KDGKTVRVVS
       670        680        690        700        710        720
FVCWELFDEQ SDEYKESVLP SDVSARVSIE AASTFGWGKI VGGKGKSIGI NSFGASAPAP
       730        740
LLYKEFGITV EAVVDAAKSF F
```

Figure S5. The analysis of key amino acid sites. Metal binding: +; Binding site: *; Important for catalytic activity site: #.

Table S1. The primers for identification.

Spot ID	Gene Name	Gene No.	Seeds Salk No.	Primers for TP-PCR identification	Primers for Q-PCR identification
435	*PRK*	AT1G32060	SAIL_881_A12	LP 5'-GCAAACACCATGACTCGAGAG-3'<br>RP 5'-AAAACAGATGGCAATTTGCAG-3'	F 5'-ACCATCTCGTGGATTCCTTGC-3'<br>R 5'-CTCGTCCAGTCTATCAAATTGTCC-3'
619	*ATTKL1*	AT3G60750	SAIL_58D02	LP 5'-TCCAACGGTCATATTTGATCC-3'<br>RP 5'-GATTCCTTGTCCAAGAGGACC-3'	F 5'-TACCTGAGAAAGCACCACCG-3'<br>R 5'-CACCCTTTTCCACTCCTTCG-3'
431	*CHLI1*	AT4G18480	SALK_132265	LP 5'-AAATGGGTGGTTTACCAATCC-3'<br>RP 5'-TCTGGTTGGAATACGGTTGAG-3'	F 5'-TAGGGACGGTTAGAGATGCTGATT-3'<br>R 5'-TCCTGCTCGGTTTTGTAAGTGT-3'
26	*ATVHA-C*	AT1G12840	SALK_072056	LP 5'-CTGCGAAATTGAAACCAAAAC-3'<br>RP 5'-ATCCTCTTGCTCCTCTTCAGC-3'	F 5'-GCTTCTTCGTTATGGAATCG-3'<br>R 5'-CTCCTTCAGAGGTGACATTGT-3'
146	*VPS25*	AT4G19003	SALK_010439C	——	F 5'-CGTGCGGAGTGGTTAGATA-3'<br>R 5'-AGACAGAGAACTTGACGCCT-3'
11	*ADK2*	AT5G03300	SALK_000565C	——	F 5'-TGAGTCGGCACCTACTGGAA-3'<br>R 5'-TCAGGCGATACTGTGAGGAAGA-3'
108	*ATELF5A-2*	AT1G26630	SALK_020969C	——	F 5'-ACCTATCCTCAATCAGCCG-3'<br>R 5'-CGAATCCAAGCCTCATCT-3'
192	*DHAR2*	AT1G75270	SALK_026089C	——	F 5'-TTCTCGGCGACTGTCCGTTTA-3'<br>R 5'-AGCCACCCATTTGCCATCA-3'
——	*LBb1*	——	——	5'-GCGTGGACCGCTTGCTGCAACT-3'	——
——	*LB2*	——	——	5'-<br>GCCTTTTCAGAAATGGATAAATAGCCTTG<br>CTTCC-3'	——
——	*ACTIN*	AT2G37620	——	——	F 5'-GAATCGCCGACAGAATGA-3'<br>R 5'-TACTGAGGGAGGCCAAGA-3'
